# M Protein from Dengue virus oligomerizes to pentameric channel protein: *in silico* analysis study

**DOI:** 10.5808/gi.23035

**Published:** 2023-09-27

**Authors:** Ayesha Zeba, Kanagaraj Sekar, Anjali Ganjiwale

**Affiliations:** 1 Department of Life Sciences, Bangalore University, Bangalore, Karnataka 560056, India; 2 Laboratory for Structural Biology and Bio-computing, Computational and Data Sciences, Indian Institute of Science, Bangalore, Karnataka 560012, India

**Keywords:** Dengue virus, inhibitors, M protein, residue interaction analysis, viroporin

## Abstract

The Dengue virus M protein is a 75 amino acid polypeptide with two helical transmembranes (TM). The TM domain oligomerizes to form an ion channel, facilitating viral release from the host cells. The M protein has a critical role in the virus entry and life cycle, making it a potent drug target. The oligomerization of the monomeric protein was studied using *ab initio* modeling and molecular dynamics simulation in an implicit membrane environment. The representative structures obtained showed pentamer as the most stable oligomeric state, resembling an ion channel. Glutamic acid, threonine, serine, tryptophan, alanine, isoleucine form the pore-lining residues of the pentameric channel, conferring an overall negative charge to the channel with approximate length of 51.9 Å. Residue interaction analysis for M protein shows that Ala94, Leu95, Ser112, Glu124, and Phe155 are the central hub residues representing the physicochemical interactions between domains. The virtual screening with 165 different ion channel inhibitors from the ion channel library shows monovalent ion channel blockers, namely lumacaftor, glipizide, gliquidone, glisoxepide, and azelnidipine to be the inhibitors with high docking scores. Understanding the three-dimensional structure of M protein will help design therapeutics and vaccines for Dengue infection.

## Introduction

The Dengue virus (DENV) is the most critical human pathogen among all arboviruses. Approximately 50–100 million DENV infections occur yearly in tropical and subtropical regions, where more than 2.5 billion people are at risk (nearly a third of the global population) [1]. DENV is one of the most widely distributed flaviviruses with four known serotypes. It is highly restricted in their natural vertebrate host range, generally utilizing primates as their amplification and reservoir hosts. The mosquito vector *Aedes aegypti* is abundant, putting almost a third of the global human population at risk of infection. There are three structural proteins in the mature DENV virion (capsid protein C, membrane protein M, and envelope protein E). At the same time, the immature intracellular virus also contains prM protein, a precursor of M. The gene order for the structural proteins from the 50 termini of the DENV genome is C–prM/M–E. The viral particles comprise an outer glycoprotein shell and an internal host-derived lipid bilayer encapsulating the RNA/protein core consisting of genome RNA and capsid protein C [2].

Replication of the viral genome primarily occurs in the cytoplasm of infected cells. The incoming viral RNA is initially translated into a polyprotein and then directed to the endoplasmic reticulum (ER). NS1 and the ectodomains of prM and E are translocated into the lumen of the ER, while the C, NS3, and NS5 proteins are localized to the cytoplasm. NS2A/B and NS4A/B remain predominantly transmembrane proteins. Processing this polyprotein is fundamental before viral RNA replication can proceed.

The glycoprotein shell has a well-defined structure that includes 180 copies of envelope protein E and membrane protein pre/M. The DENV particles can exist in mature and immature forms that are morphologically different (immature particles are 'spiky', whereas mature particles are 'smooth'). The immature virion has 60 trimeric spikes extending from the particle surface and 90 heterodimers of prM. In contrast, protein E of the mature virion forms 90 homodimers lying flat against the viral surface, creating a ‘smooth’ protein shell. The conformational changes in the viral E and prM/M proteins lead to the transition between these two forms of the viral particle, with the predominant role being played by structural changes in protein E. The 'pr' peptide controls the premature fusion of the immature virions with the host membrane and is cleaved in the mature particle. M acts as a transmembrane protein beneath the E protein shell in the mature particle. The DENV prM glycoprotein consists of 166 amino acids with the N-terminal' pr' polypeptide (residues 1–91) followed by the ectodomain (residues 92–130) and the C-terminal transmembrane region (residues 131–166) [3-5]. The cleavage of prM to M, which follows two basic amino acid residues, requires an acidic environment. Following cleavage and fusion of the vesicle with the plasma membrane, the amino-terminal end of prM (non-M) is released into the medium [6].

The C-terminal peptide of the M protein from the DENV forms ion channels in lipid bilayer membranes and forms ion channels in lipid bilayers or cells [7]. These virus ion channels are called 'viroporins', similar to porins in bacterial membranes with large conductance, non-selective channels. However, the virus ion channels are quite different. They can select between cations and anions and may have a low conductance [7]. Across different viruses, these virus-coded channel proteins vary significantly in their structure and are known to perform multiple functions during the virus life cycle. M2 proton channel of influenza A virus is the prototype viroporin with provenance as an antiviral drug target. Literature shows the rapid expansion of viroporin family to include significant human pathogens like severe acute respiratory syndrome coronavirus 2 (SARS-CoV-2), human immunodeficiency virus type 1, picornaviruses, alphaviruses and paramyxovirus [8,9]. Structurally viroporins are 50–100 amino acids long and comprise one, two, or three potential transmembrane domains. Small length requires them to oligomerize to form intact pores across the membrane. Examples range from tetrameric M2 proton channel (PDB ID 2RLF, 3BKD) from Influenza to heptameric channel from hepatitis C virus (PDB ID 2M6X).

DENV M protein is localized to the ER, and has the propensity to self-assemble into higher-order oligomers forming ion channels [10]. Similarly, in the Zika virus, the mature M protein residing within the mature ZIKV virion acts as an ion channel with a critical function during virus entry. Thus, M channels represent a potential new target for antiviral therapies that could limit disease severity and engender prophylactic use to disrupt transmission [11]. M protein was previously proposed to function as a viroporin based primarily on its size and hydrophobicity. Yet, conclusive evidence for an outlined role within flavivirus life cycles has been lacking.

Our study proposes three-dimensional molecular models of the M protein of the DENV which is a potential therapeutic and vaccine target. The ectodomain region was used as a monomer from 92 to 130, followed by the transmembrane (TM) region from 131 to 166 amino acid residues. Oligomerization of the protein was studied using molecular dynamics (MD) simulations in a dodecyl phosphocholine (DPPC) bilayer mimicking the biological membrane. The binding pocket of the stable oligomer selected in the study was further characterized using virtual screening and docking of the various ion channel inhibitors. This manuscript demonstrates the application of M protein as a drug target for developing novel therapies against Dengue infections.

## Methods

### Sequence alignment and phylogenetic tree

The amino acid sequence of the M protein of DENV for different strains was retrieved from the Uniprot database. M protein sequences of related flavivirus families were used to generate the multiple sequence alignment using ClustalW (https://www.genome.jp/tools-bin/clustalw). Subsequently, phylogenetic trees were constructed based on multiple sequence alignment as an input with neighbor joining method using simple phylogeny [12,13].

Secondary structure was predicted using Phyre 2 webserver (http://www.sbg.bio.ic.ac.uk/phyre2) and alpha fold v2.3.1 [14,15]. Alongside, transmembrane regions were also predicted using deep TMHMM [16].

### Molecular modeling

The first step in molecular modeling includes template search. No suitable template with significant homology was found using PDB BLAST and SwissModel for the M protein of DENV. Subsequently, models were generated by abinitio and artificial intelligence (AI) modeling algorithms. RoseTTA fold was used to develop the abinitio model [17]. It is the most accurate method provided by the server using a deep learning-based modeling method. Another AI method, Alphafold, was used for molecular modeling [15,18]. The output models were then assessed using ERRAT, VERIFY, PROVE, and PROCHECK (https://saves.mbi.ucla.edu/). Further, the models were evaluated in the ModEval server and the DOPE (Discrete optimized protein energy) score was obtained along with the GA341 score, z-surf, z-pair, and z-combi [19-21]. The GA341 score, along with z-pair, z-surf, and z-combi are based on the statistical potentials and a model score greater than the predetermined cutoff of 0.7 is considered reliable [19]. DOPE score represents atomic distance-dependent statistical potential, and a model with DOPE score of less than 0 is considered reliable.

Oligomers with the dimer, trimer, tetramer, pentamer, and octamer symmetry of M protein were generated using Symmdock [22]. The algorithm predicts the Cn symmetry type complex with rotational symmetry of order n about the symmetry axis. The method outputs a list of complexes that fulfill the cyclic symmetry constraints. The top 10 models out of 100 models were chosen for further analysis.

### MD simulations

Monomer, dimer, and trimer models obtained do not form the channel-like structure. All the higher-order oligomers starting from tetramer to octamer were evaluated for flexibility and stability in the membrane environment using GROMACS 2022 [23]. Each oligomer was oriented and packed in a DPPC bilayer of 128 phospholipids. GROMOS96 53A6 force field was modified to incorporate lipid parameters [24,25]. Each system was solvated with water molecules and neutralized with sodium and chloride ions followed by energy minimization. The NVT and NPT equilibration were performed coupling with 323K temperature and 1 bar pressure. Finally, the production simulation for 50 to 200 ns was performed till the root mean square deviation (RMSD) of the backbone of the polypeptide became stable with reference to the starting structure. Flexibility was measured using root mean square fluctuation (RMSF) and compactness of the structure was measured using radius of gyration (Rg).

### Pore analysis and docking

Size, shape, and identification of the protein main axis and analysis of pore features were characterized using MOLE2.5 toolkit [26]. Chemical structures of 165 ion channel drugs on the market were downloaded from the ion channel library with known mechanisms of action such as sodium/potassium channel inhibitors, calcium channel inhibitors, and multichannel blockers, etc. (https://www.ionchannellibrary.com/ion-channel-drugs/). Structure-based virtual screening was performed using AutoDock Vina compiled with PyRX tool [27-29]. The active site for the M protein was characterized using the active site prediction server (http://www.scfbio-iitd.res.in/dock/ActiveSite.jsp). The protein was imported into PyRx, and the binding pocket search space encompassed the whole of the pentameric channel protein with center (x,y,z) = (76.99, 77.001, 76.98), dimensions (x,y,z) = (25, 25, 25) Å and the value of exhaustiveness was set to its default.

The docked compounds were ranked and selected based on the highest binding affinity. The binding pocket for the best-scoring compound, lumacaftor was further characterized using MD simulations with GROMACS to confirm the binding mode and the stability. Topologies for the lumacaftor and the M protein were generated, and the CHARMM36 forcefield was applied as the initial step [30,31]. The MD simulation up to 60 ns was carried out as described in section 2.3. The trajectories obtained were further analyzed using GROMACS utilities.

## Results

### Sequence analysis and alignment

**Fig. 1.**
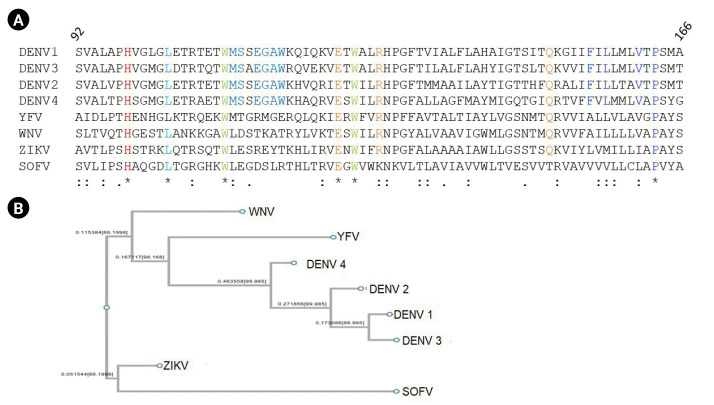
(A) M Protein sequence alignment using ClustalW. (B) Phylogenetic tree of M protein of members of the flavivirus family.

The amino acid sequence for M protein of DENV, along with the sequences of M proteins reported for other flaviviruses, were retrieved from the Uniprot database. A total of eight sequences from four different strains of DENV along with West Nile (WNV), Zika (ZIKV), Tick borne encephalitis virus Sofjin strain (SOFV), and yellow fever virus (YFV) were retrieved (accession IDs: DENV1 [P17763], DENV2 [P29990], DENV3 [Q6YMS4], DENV4 [P09866], WNV [P06935], ZIKV [A0A024B7W1], SOFV [P07720], and YFV [Q9YRV3]). The sequences were analyzed for conservation of amino acids using multiple sequence alignment of M Protein across the flavivirus family from amino acid residue 92 to 166 representing M protein. DENV1 has 64% identity with DENV2, 57% identity with DENV3. A total of 36 amino acids out of 75 are conserved across the 4 DENV strains. The region 92 to 130 represent the ectodomain, and 131 to 166 represent the C-terminal transmembrane region of the polypeptide. As shown in Fig. 1A, His99, Leu103, Trp110, Glu124, Trp126, and Pro163 are conserved across M protein of the representative members of the flavivirus family. The phylogenetic tree constructed based on the sequence identity and similarity shows two major branches representing ZIKV and SOFV on the same clad and all other viruses including four strains of DENV, YFV, and WNV on the other (Fig. 1B).

M Protein sequence for DENV1 strain with accession ID P17763, was studied using the Phyre 2 server which showed 72% helix and 41% transmembrane region. DeepTMHMM server was used to locate transmembrane regions in the M Protein sequence, resulting in two transmembrane helices (Supplementary Figs. 1 and 2).

### Molecular modeling and MD simulation

The three-dimensional models for M Protein were generated using RoseTTA fold. The algorithm generated five models with alpha-helices, transmembrane helices, disorder prediction, and error estimation of every amino acid residue. Alpha fold prediction shows higher per residue confidence metric called pLDDT scores for residues 20 to 60, indicating higher accuracy (Supplementary Figs. 3 and 4). Robetta model1 shows a negative DOPE score with GA341 score of 1, more significant than the predetermined cut-off value of 0.7. Robetta model1 has an overall quality factor of 84.104 with all the residues within the allowed region of the Ramachandran plot (Supplementary Table 1, Supplementary Fig. 5).

SymmDock is one of the geometry-based algorithms for predicting a cyclically symmetric complex, given the structure of its asymmetric unit. The given asymmetric unit was a monomer of M Protein for which cyclically symmetric transformations of a given order 2, 3, 4, 5, 6, 7, and 8 were generated. Hence, the resulting oligomers were dimer, trimer, tetramer, pentamer, hexamer, heptamer, and octamer in symmetry (Supplementary Fig. 6).

**Fig. 2.**
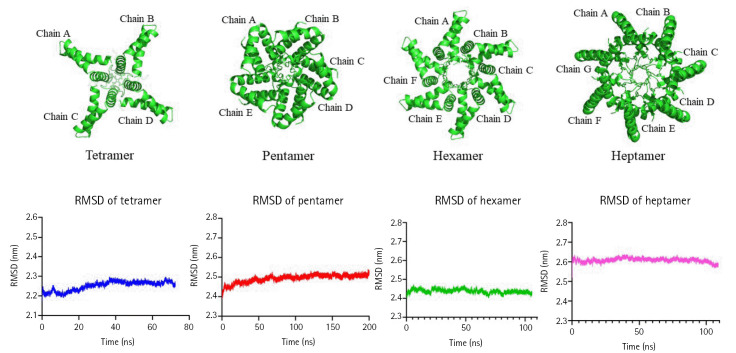
Molecular dynamics simulation for higher-order oligomers for M protein Dengue virus in dipalmitoylphosphatidylcholine bilayer with the root mean square deviation (RMSD) plots using GROMACS.

**Fig. 3.**
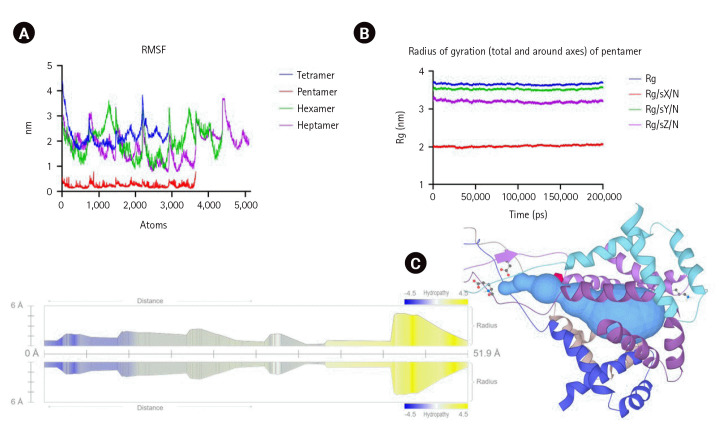
(A) Molecular dynamics simulation root mean square fluctuation (RMSF) fluctuation for atoms in higher-order oligomers. Pentamer shows the least RMSF. (B) The overall radius of gyration for the pentamer. (C) Binding pocket analysis of pentameric M protein channel with MOLE2.5 kit.

The MD simulation of the oligomer-DPPC membrane system was performed with GROMACS. DPPC is one of the most abundant lipids in the biological membranes and is experimentally and computationally well-characterized system. The octamer system was unstable during the MD run and was eliminated from further analysis. The trajectory analysis of tetramer, pentamer, hexamer, and heptamer was performed for three different parameters: RMSD for the backbone atoms with reference to the starting conformation, RMSF indicating standard deviation of atomic positions of C-alpha atoms and the Rg values indicating the compactness of the structure (Figs. 2 and 3). Pentameric form shows the least RMSF and stable RMSD values with a total Rg of 2.2 ± 0.021 nm. Pentamer was used for further analysis and characterization.

### Pore analysis and docking

The pore analysis using the MOLE2.5 kit shows a channel length of 51.9Å lined by Trp, Ile, Leu, Ser, and Glu amino acids conferring an overall negative charge to the channel. Molecular docking effectively studies the interaction between ligands and biological macromolecules. The binding capacity of standard 165 ion channel inhibitors from the ion channel library to the pentameric form of M protein was assessed via molecular docking. AutoDock Vina, and MGL autodock tools were run with their default settings [29]. The protein backbone remained rigid, the ligands were flexibly treated, and the docking box (76.99 Å × 77.001 Å × 76.98 Å) covered as many protein surfaces as possible during the docking process. A grid spacing of 0.375 Å was used to enclose the active site. Lamarckian genetic algorithm was used for docking calculation, and the search parameter was set to 100 times. Subsequently, the docking model with the lowest binding energy of ligand in the binding pocket of protein was selected as the best model. The Vina score was used as the predictive affinity of peptide binding to M protein (calculated in kcal/mol). Out of 165 ion channel inhibitors, monovalent ion channel inhibitors like chloride, potassium, and sodium predominantly bind the M protein pentamer. The five best compounds (lumacaftor, glipizide, gliquidone, glisoxepide, and azelnidipine) showed high binding free energy scores (–9.6 to –8.4 kcal/mol).

**Fig. 4.**
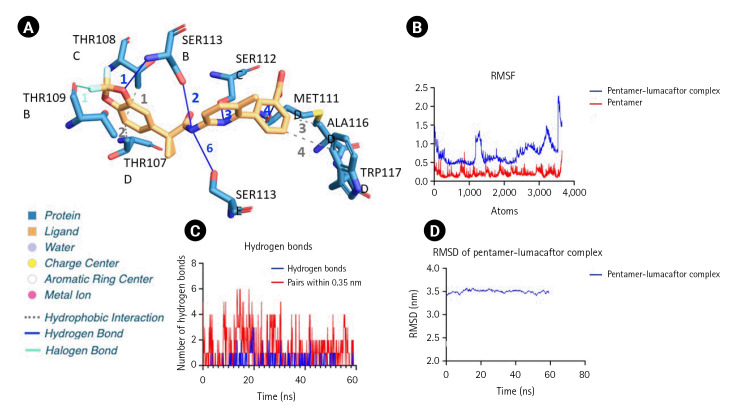
(A) Binding pocket analysis of pentameric M protein with lumacaftor, a chloride channel inhibitor. (B) Root mean square fluctuation (RMSF) of M protein pentamer-lumacaftor complex. (C) Analysis of hydrogen bonds in pentamer-lumacaftor complex. (D) Root mean square deviation (RMSD) of pentamer-lumacaftor complex.

Lumacaftor exhibited the lowest energy value of –9.6 kcal/mol, suggesting that this ion channel inhibitor could have the highest binding affinity for the pentameric form of M protein. The binding pocket analysis using protein-ligand interaction profiler shows hydrophobic interactions involving Thr107, Ala116, and Trp117 residues and the hydrogen bond network involving Ser112 and Met111 of the ectodomain region (Fig. 4) [32]. As observed in multiple sequence alignment, these binding pocket residues are conserved across the DENV strains. The binding pocket interactions for the top scoring ion channel ligand, lumacaftor is as shown in Fig. 4 and summarized in Supplementary Table 2. In addition, the MD simulation shows lumacaftor stably binding to pentameric M protein with RMSF fluctuations in the range of 0.86 ± 0.30 nm.

## Discussion

M protein model reported in this study demonstrates characteristics analogous to viroporins, and ion channel inhibitors bind predominantly to the ectodomain of the channel stabilized by hydrophobic interactions and hydrogen bond network. The channels are selective for monovalent ions, as indicated by the screening of 165 ion channel inhibitors. Our study shows that the M protein is the pentameric channel protein with an overall negative charge and approximate length of 51.9Å. The overall negative charge on the channel might be responsible for the binding selectivity of monovalent cations.

Viroporins are found across the virus family and are known to play an important role in virus replication and pathogenesis. A pore-forming alpha-helical transmembrane domain with a cytoplasmic and N-terminal domain characterizes viroporins. Based on the number of transmembrane domains viroporins are classified as class I (with one TM domain) and class II (with two TM domain) [9]. Based on our analysis M protein of DENV belongs to class I viroporins with single TM domain. Other examples of class I viroporins include M2 proton channel, viral protein u (Vpu), small hydrophobic protein (SH protein) from human respiratory syncytial virus, etc.

**Fig. 5.**
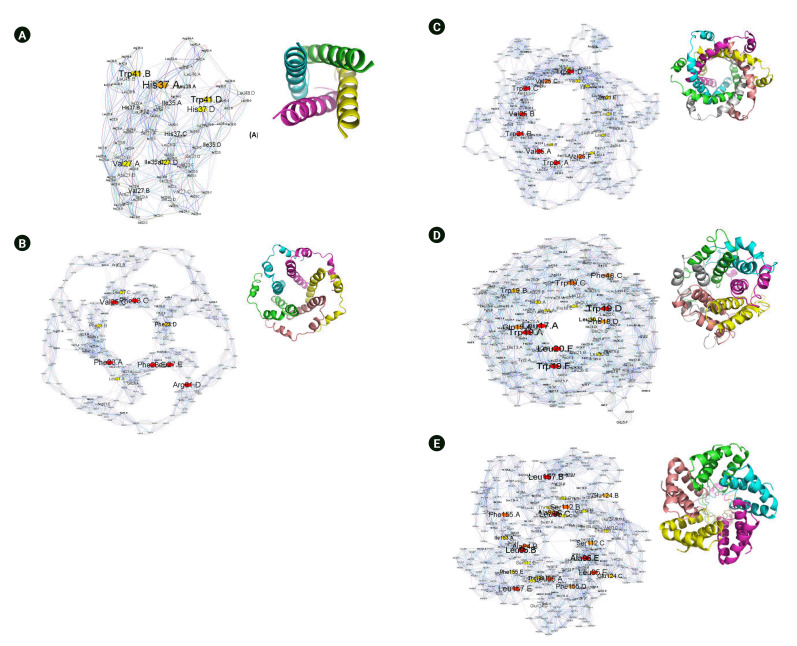
Residue interaction network representing residue centrality analysis of PDB ID: 6BKK-tetrameric M2 proton channel of influenza virus (A), PDB ID: 5X29-pentameric E channel proteins of SARS CoV2 (B), PDB ID: 2M6X- hexameric P7 channel protein of hepatitis C virus (C), hexameric 6K channel protein model of Chikungunya virus (D), and pentameric M protein model of DENV (E).

It was of interest to compare the different viroporin oligomers as a first step to understanding their function. Oligomerization of the protein is related to its primary amino acid sequence. We performed residue interaction network analysis to find the relationship between protein sequence and to identify the network's essential hub residues using structureViz2 and RINAnalyzer app in cytoscape [33-36]. Fig. 5 shows the residue interaction network of tetrameric M2 proton channel of infleunza virus (PDB ID: 6BKK [37]), pentameric E protein of SARS-CoV-2 (PDB ID: 5X29 [38]), hexameric P7 channel protein of hepatitis C virus (PDB ID: 2M6X [39]), hexameric 6K channel protein model of Chikungunya virus [40] and pentameric M protein channel of DENV. The residue centrality analysis (RCA) shows that the M2 proton channel has Val27, Ile35, His37, and Trp41 as the hub residues. The aromatic residues Trp41 and His37 have been reported to change the conformation of the M2 proton channel with the change in pH [41], indicating a possible important role of central residues. Similarly, the pentameric E channel protein of SARS-CoV-2 is a 75 amino acid long single-pass TM protein. Deletion of E protein in recombinant coronavirus has shown reduced viral titres and maturation [42]. The RCA for E protein shows Phe23, Val25, Phe28, Leu27, and Arg61 as the hub amino acid residues. E protein has also been shown to interact with Amantadine, the M2 proton channel blocker, indicating a similar plausible role of aromatic Phe rings in the functioning of the channel. The RCA analysis of hexameric P7 channel shows Trp21 and Val25 to be the central residues in the network, indicating critical residues required for interaction. The hexameric 6K channel shows Leu17, Phe18, Trp19, and Leu20 as the central hub residue. In contrast, the pentameric M protein of DENV, RCA analysis shows Ala94, Leu95, Ser112, Glu124, and Phe155 are the central hub residues (Fig. 5A–E).

As the diverse functions of viroporins continue to be identified, it has been shown that their function is essential to the virus life cycle making them potent drug targets. The pentameric channel model of the M protein proposed in this study is the first step in understanding the function and the development of novel therapeutic strategies for DENV.

## Supplementary Materials

Supplementary Table 1. Tabulated results of models of M protein using ModBase evaluation with its DOPE score, GA341, z-pair, z-surf, and z-combi

Supplementary Table 2. Binding pocket analysis of top 4 ion channel ligands from ion channel library with pentameric M protein model

Supplementary Fig 1. (A) Secondary structure and disorder prediction indicating alpha helix and transmembrane helix from phyre2 server. (B) The amino acid residues of M protein that are present in the transmembrane region have been indicated.

Supplementary Fig. 2. The predicted topology of transmembrane regions in M protein.

Supplementary Fig. 3. (A) Alpha helices and transmembrane helices and disorder prediction of M protein by Rosetta Fold server. (B) Monomeric model generated by Rosetta Fold. (C) Error estimation of every amino acid residue.

Supplementary Fig. 4. Molecular model predicted by Alpha Fold.

Supplementary Fig. 5. (A) Ramachandran plot analysis (B) Quality factor of the M protein Robetta Model 1.

Supplementary Fig. 6. Structural arrangements of Dengue virus 1 small envelope protein M 1–75 (monomer to octamer).
