# Supplementary material for: M Protein from Dengue virus oligomerizes to pentameric channel protein: in silico analysis study
Source: Genomics Inform. 2023 Sep 27;21(3):e41. doi: 10.5808/gi.23035 (PMC10584644; doi:10.5808/gi.23035)
Supplement: Supplementary Table 1. — Tabulated results of models of M protein using ModBase evaluation with its DOPE score, GA341, z-pair, z-surf, and z-combi [file gi-23035-Supplementary-Table-1.pdf]

SUPPLEMENTAL INFORMATION

Supplementary Table 1. Tabulated results of models of M protein using ModBase evaluation with its DOPE score, GA341, z-pair, z-surf, and z-combi

Model	DOPE	GA341	z-pair	z-surf	z-combi
Alphafold (1-75)	0.082	0.287	–2.758	–1.793	–3.095
Robetta 1 (1-75)	–1.870	1.000	–4.473	–3.496	–5.837
Robetta 2 (1-75)	–1.637	1.000	–4.513	–4.074	–6.624
Robetta 3 (1-75)	–1.558	1.000	–5.243	–3.634	–6.454
Robetta 4 (1-75)	–1.397	1.000	–4.430	–3.142	–5.447
Robetta 5 (1-75)	–1.513	1.000	–4.353	–3.234	–5.647
