# Supplementary material for: M Protein from Dengue virus oligomerizes to pentameric channel protein: in silico analysis study
Source: Genomics Inform. 2023 Sep 27;21(3):e41. doi: 10.5808/gi.23035 (PMC10584644; doi:10.5808/gi.23035)
Supplement: Supplementary Table 2. — Binding pocket analysis of top 4 ion channel ligands from ion channel library with pentameric M protein model [file gi-23035-Supplementary-Table-2.pdf]

Supplementary Table 2. Binding pocket analysis of top 4 ion channel ligands from ion channel library with pentameric M protein model

Ligand	Mechanism of action	Binding affinity score for docking from auto dock vina	Hydrogen bond	Hydrophobic bond	Others
Lumacaftor	CFTR modulator	–9.6	Met 111, Ser 113	Thr 107, Thr 109, Ala 116, Trp 117	Thr 109 (halogen bond)
Glipizide	Kir6.2 inhibitor	–8.7	Thr 109, Ser 113	Thr 109, Ala 116, Trp 117	
Gliquidone	Kir6.2 blocker	–8.3	Ser 113	Thr 107, Ala 116, Trp 118	
Azelnidipine	Cav1.x blocker	–8.1	Ser 113	Thr 107, Thr 109, Ala 116, Trp 117	
