# Supplementary material for: M Protein from Dengue virus oligomerizes to pentameric channel protein: in silico analysis study
Source: Genomics Inform. 2023 Sep 27;21(3):e41. doi: 10.5808/gi.23035 (PMC10584644; doi:10.5808/gi.23035)
Supplement: Supplementary Fig 1. — (A) Secondary structure and disorder prediction indicating alpha helix and transmembrane helix from phyre2 server. (B) The amino acid residues of M protein that are present in the transmembrane region have been indicated. [file gi-23035-Supplementary-Fig-1.pdf]

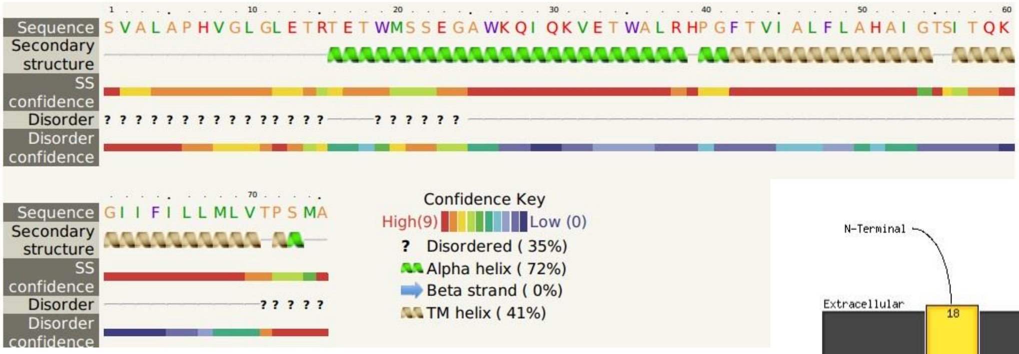

B

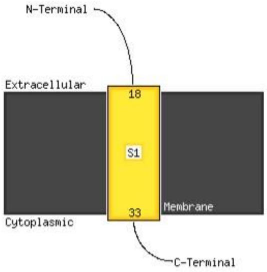

(A) (B)

.

Supplementary Fig. 1. (A) Secondary structure and disorder prediction indicating alpha helix and transmembrane helix from phyre2 server. (B) The amino acid residues of M protein that are present in the transmembrane region have been indicated.
