# Supplementary material for: M Protein from Dengue virus oligomerizes to pentameric channel protein: in silico analysis study
Source: Genomics Inform. 2023 Sep 27;21(3):e41. doi: 10.5808/gi.23035 (PMC10584644; doi:10.5808/gi.23035)
Supplement: Supplementary Fig. 2. — The predicted topology of transmembrane regions in M protein. [file gi-23035-Supplementary-Fig-2.pdf]

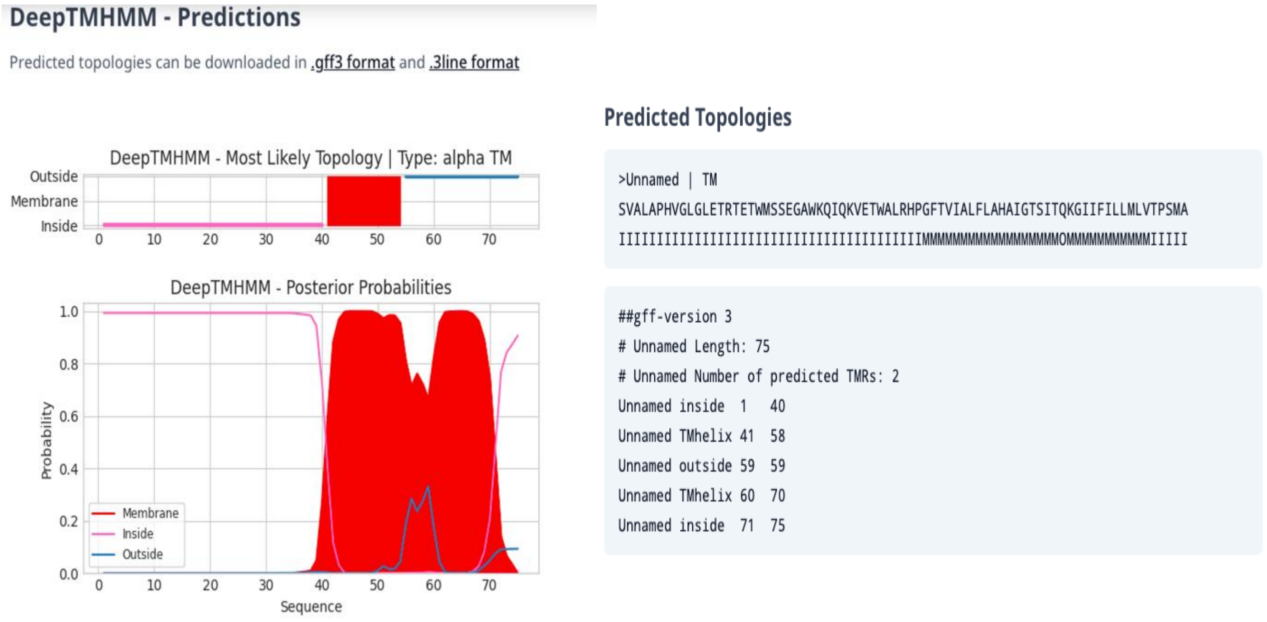

Supplementary Fig. 2. The predicted topology of transmembrane regions in M protein.
