# Supplementary figures and images for: M Protein from Dengue virus oligomerizes to pentameric channel protein: in silico analysis study
Source: Genomics Inform. 2023 Sep 27;21(3):e41. doi: 10.5808/gi.23035 (PMC10584644; doi:10.5808/gi.23035)

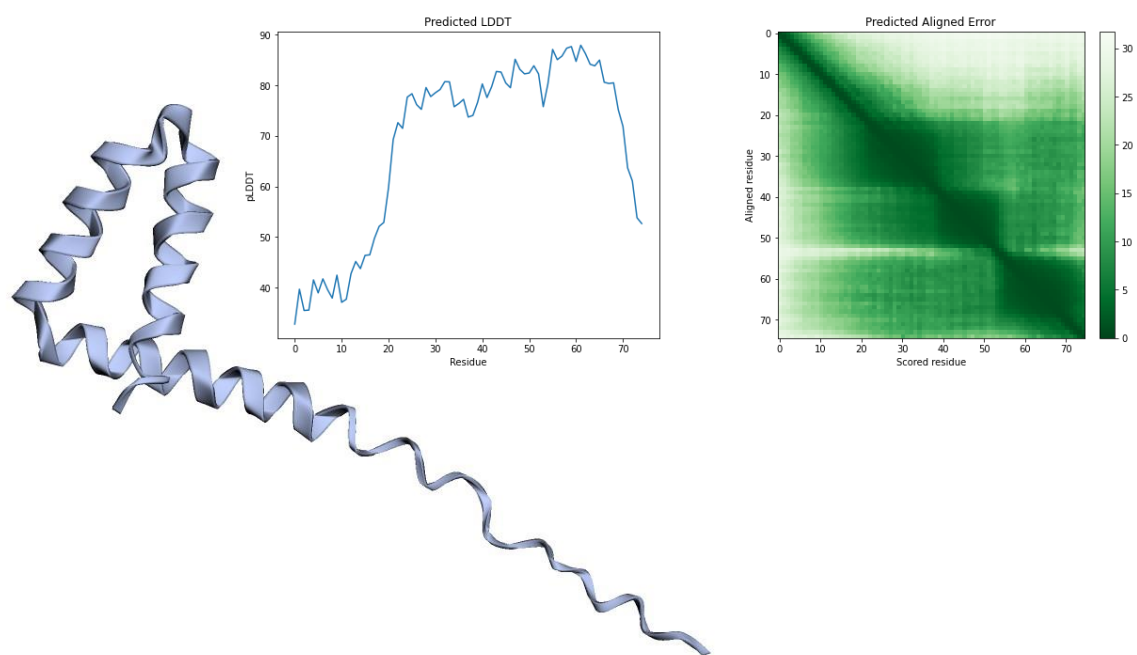

**Supplementary Fig. 4.** Molecular model predicted by Alpha Fold.

Supplement: Supplementary Fig. 4. — Molecular model predicted by Alpha Fold. [file gi-23035-Supplementary-Fig-4.pdf]

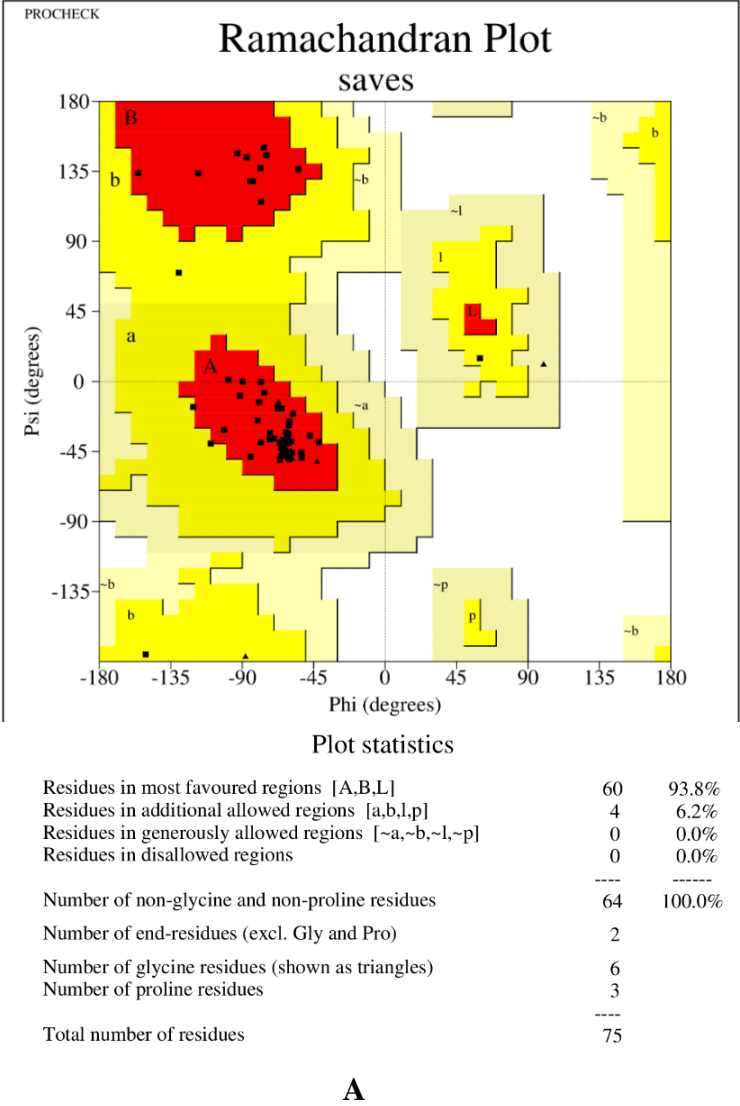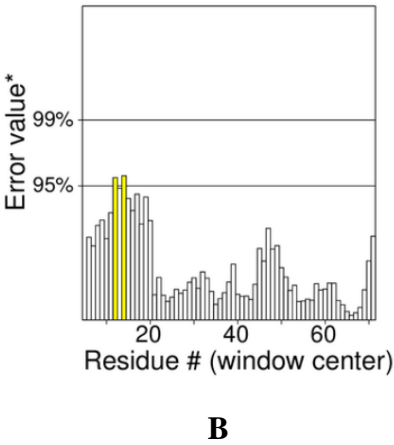

Supplementary Fig. 5.

Supplement: Supplementary Fig. 5. — (A) Ramachandran plot analysis (B) Quality factor of the M protein Robetta Model 1. [file gi-23035-Supplementary-Fig-5.pdf]
