# Supplementary material for: M Protein from Dengue virus oligomerizes to pentameric channel protein: in silico analysis study
Source: Genomics Inform. 2023 Sep 27;21(3):e41. doi: 10.5808/gi.23035 (PMC10584644; doi:10.5808/gi.23035)
Supplement: Supplementary Fig. 6. — Structural arrangements of Dengue virus 1 small envelope protein M 1–75 (monomer to octamer). [file gi-23035-Supplementary-Fig-6.pdf]

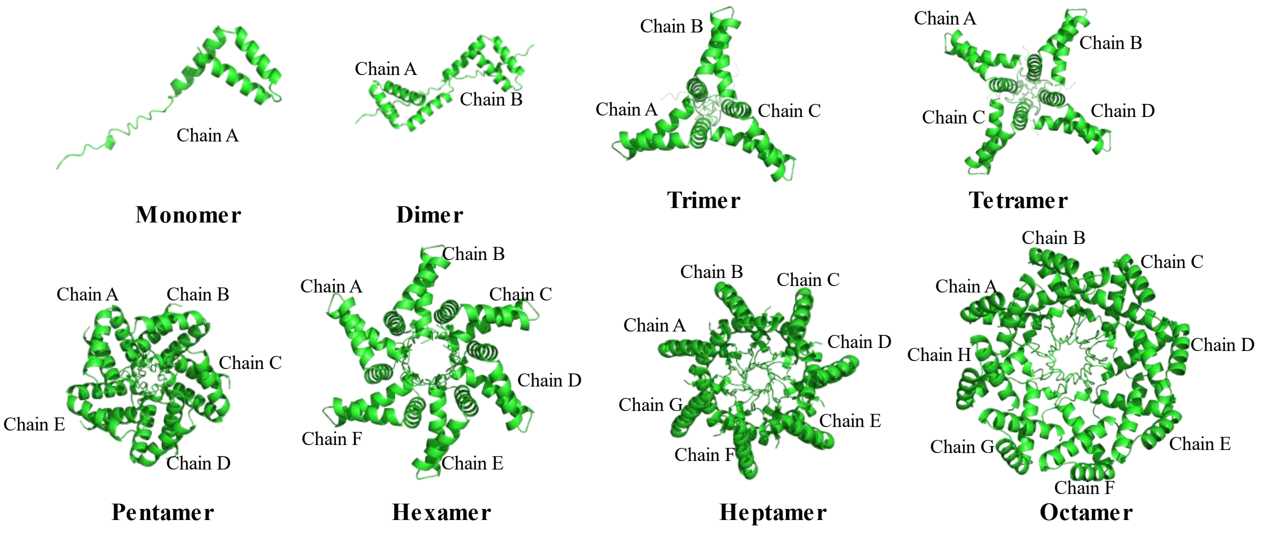

Supplementary Fig. 6. Structural arrangements of Dengue virus 1 small envelope protein M 1–75 (monomer to octamer).
