# Supplementary material for: M Protein from Dengue virus oligomerizes to pentameric channel protein: in silico analysis study
Source: Genomics Inform. 2023 Sep 27;21(3):e41. doi: 10.5808/gi.23035 (PMC10584644; doi:10.5808/gi.23035)
Supplement: Supplementary Fig. 3. — (A) Alpha helices and transmembrane helices and disorder prediction of M protein by Rosetta Fold server. (B) Monomeric model generated by Rosetta Fold. (C) Error estimation of every amino acid residue. [file gi-23035-Supplementary-Fig-3.pdf]

```
              1   .    10   .    20   .    30   .    40   .    50   .    60   .    70   .
  sequence: SVALAPHVGLGLETRTETWMSSEGAWKQIQKVETWALRHPGFTVIALFLAHAIGTSITQKGIIFILLMLVTPSMA
deepconcnf: -EEE------------------HHHHHHHHHHHHHHHH-HHHHHHHHHHHHHH---HHHHHHHHHHHHHH-----
   psipred: -EEE------------------HHHHHHHHHHHHHHHH-HHHHHHHHHHHHHH----HHHHHHHHHHHHH-----
   spider3: ----------------------HHHHHHHHHHHHHHH--HHHHHHHHHHHHHH---HHHHHHHHHHHHHHHHHH-
  disopred: --------DDDDDDDDDD--------------------------------------------------------D
     tmhmm: -----------------------------------------------TTTTTTTTTTTTTTTTTTTTTTT-----
```

(A)

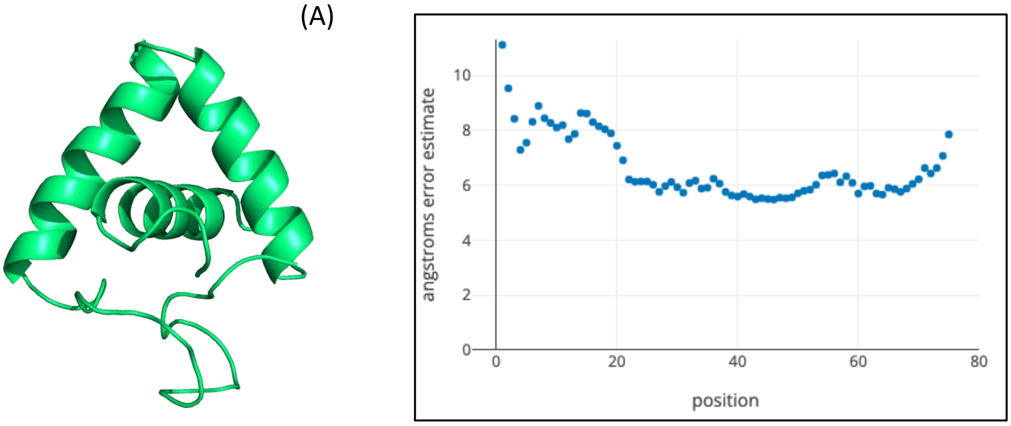

(B) (C)

Supplementary Fig. 3. (A) Alpha helices and transmembrane helices and disorder prediction of M protein by Rosetta Fold server. (B) Monomeric model generated by Rosetta Fold. (C) Error estimation of every amino acid residue.
